# Effects of Chinese herbal medicine in combination with mitomycin C on gastric cancer cells

**DOI:** 10.1186/s40364-014-0026-8

**Published:** 2014-12-24

**Authors:** Che-Chang Kuo, Jian-Jung Chen, James Y Tsai, Chung-Tsen Hsueh

**Affiliations:** Department of Chinese Medicine, St. Joseph Hospital, Kaohsiung, Taiwan. School of Chinese Medicine for Post-Baccalaureate, I-Shou University, Kaohsiung, Taiwan; Department of Chinese Medicine, Taichung Tzu Chi General Hospital, Taichung, Taiwan; Division of Medical Oncology and Hematology, Loma Linda University, Loma Linda, California USA

**Keywords:** Gastric cancer, Chinese herbal medicine, Mitomycin C, Cytotoxicity, Apoptosis

## Abstract

Chinese herbal medicine (CHM) is frequently used by cancer patients in Chinese community. It remains largely unknown about the interaction between CHM and chemotherapeutic agents. Herein, we evaluated 3 commonly used CHM formulas for cancer patients: Bu-Zhong-Yi-Qi-Tang (BZYQT), Bao-Yuan-Tang (BYT), and Ju-Yuan-Jian (JYJ). We examined the effects of these 3 formulas in human gastric cancer cells MKN-74, in terms of cytotoxicity and apoptosis induction when used alone or in combination with mitomycin C (MMC). Cytotoxicity was determined by tetrazolium dye colorimetric assay. The 10% inhibitory concentration of CHM was used in this study. Cells were first exposed to CHM or phosphate buffered saline (as control) for 48 h. Then MMC at final concentration of 0.25 μg/ml was added to media for another 24-h. Among these 3 CHM formulas, BZYQT showed the most pronounced effect in augmenting MMC-induced cytotoxicity. The viability of MKN-74 cells was decreased to 43.1% when treated with BZYQT and MMC, compared to 94.9% with MMC alone. We subsequently examined apoptosis induction by quantitative florescent microscopy and single-strand DNA enzyme-linked immunosorbent assay, and found BZYQT did not enhance MMC-induced apoptosis. Our findings indicate BZYQT in combination with MMC induces cell death in gastric cancer cells via non-apoptotic mechanism. Our results provide a rationale for further investigation in the interaction of CHM and anti-cancer treatment.

## Background

There has been an increasing trend for cancer patients to take alternative medicine with standard treatment [1,2]. Alternative medicine is used by many cancer patients with intentions to improve their health and decrease the side effects from anti-cancer treatment. The most common form of alternative medicine used by cancer patients in Taiwan and China is Chinese herbal medicine (CHM) [3-5]. In Taiwan, it has been shown that more than 50% of cancer patients are taking CHM [6,7].

Qi-invigorating herbs such as Ginseng, Astragalus, Glycyrrhiza and Atractylodes are commonly prescribed CHM for cancer patients in Chinese community during their cancer treatment [8,9]. In preclinical studies, these CHM have been shown to exhibit immunomodulatory effects, and in some instances even anti-cancer activity. Patients are given these herbs in different combination formulations to reduce toxicity of cancer treatment. However, it is not known whether these CHM formulations interact with cancer treatment such as chemotherapy. In this study, we investigated the effects of CHM on gastric cancer cells, and the interaction between CHM and mitomycin C (MMC) in terms of cytotoxicity and apoptosis induction on gastric cancer cells.

## Methods

### Cell culture and drug treatment

Early-passage human gastric cancer MKN-74 cells were established and characterized as described previously [10,11]. All the cultures were maintained in standard MEM media supplemented with 100 units/ml penicillin, 100 μg/ml streptomycin and 20% heat inactivated normal calf serum (Gibco) at 37°C in a humidified atmosphere of 5% CO_2_. Cells were checked for mycoplasma contamination at least every 6 months and consistently tested negative. MMC, MTT ([3-(4,5-dimethylthiazole-2-yl)-2,5-diphenyl tetrazolium bromide] and bisbenzimide trihydrochloride (Hoechst 33258) were purchased from Sigma. Safingol was purchased from Aventi Polar Lipids. For drug treatment, exponentially growing MKN-74 cells were seeded into 96-well culture plate at 2.5 × 10^3^ cells per 200 μl of media, and allowed to adhere overnight. The combination treatment was performed with CHM given for 48 h followed by the addition of MMC for another 24 h. In the control group, phosphate buffered saline (PBS) was used instead of CHM.

### Preparation of CHM extract

Three formulations of Qi-invigorating CHM were used and obtained from Koda Pharmaceutical (Taoyuan County, Taiwan). Bu-Zhong-Yi-Qi-Tang (BZYQT) contains 10 species of medicinal plants [12]. Bao-Yuan-Tang (BYT) contains 4 species of medicinal plants including Radix Ginseng (root of Panax ginseng C.A. MEY), Radix Glycyrrhizae Uralensis (root of Glycyrrhiza uralensis FISCHER), Cortex Cinnamonomi Cassiae (cortex of Cinnamomum cassia PRESL), and Radix Astragali Membranaceus (root of Astragalus membranaceus BUNGE). Ju-Yuan-Jian (JYJ) contains 5 species of medicinal plants including Radix Ginseng (root of Panax ginseng C.A. MEY), Radix Glycyrrhizae Uralensis (root of Glycyrrhiza uralensis FISCHER), Radix Astragali Membranaceus (root of Astragalus membranaceus BUNGE), Rhizoma Cimicifugae (root and rhizome of Cimicifuga foetida L.), and Rhizoma Astractylodis Macrocephalae (Root and rhizome of Atractylodes macrocephala KOIDZUMI). The hot-water extract of CHM was prepared, and concentrated to 1 g/ml in distilled water. The extract of CHM was further dissolved in PBS with the final concentration of 100 mg/ml. The insoluble ingredients were spun down by centrifugation at 1200 × *g* for 30 min. The supernatant was sequentially passed through 0.45-μm and 0.22-μm filters for sterilization, then aliquoted and stored at −20°C.

### Cell viability assay

After drug treatment, cells were assayed for viability using MTT colorimetric method [13]. In brief, MTT (5 mg/ml) at 10% volume of culture media was added to each well, and cells were incubated for another 3 h at 37°C. Then supernant was removed, and 200 μl of acidic isopropanol was added to each well to dissolve formazan. Spectrophotometrical absorbance at 570 nm and 690 nm wavelengths was measured with a microplate reader. Background absorbance of plate at 690 nm was subtracted from the 570 nm measurement.

### Quantitative fluorescent microscopy

As previously described, the quantitative fluorescent microscopy method for apoptosis determination involves staining with Hoechst 33258 of condensed chromatin, which characterizes the cells undergoing apoptosis [14,15]. After trypsinization, adherent and non-adherent cells were washed with PBS, fixed in 3% paraformaldehyde, and then incubated at room temperature for 10 min. After removing fixative, the cells were washed with PBS, resuspended in 20 μl PBS containing 8 μg/ml of Hoechst 33258, and incubated at room temperature for 15 min. Aliquots of the cells (10 μl) were placed on glass slides coated with 3-amino-propyl-triethoxysilane, and duplicate samples of 400 cells each were counted and scored for the incidence of apoptotic chromatin condensation using an Olympus BH-2 fluorescence microscope.

### Single-strand DNA enzyme-linked immunosorbent assay (ELISA)

The apoptosis was quantitatively by a single-strand DNA ELISA kit (Roche). Briefly, 10^4^ cells were seeded in 96-well culture plate and allowed to adhere overnight. After drug treatment, the plates were centrifuged at 200 × g for 5 min and culture medium was removed. Subsequently, 200 μl of 80% methanol in PBS as fixative was added to each well, and plates were incubated at room temperature for 30 min. After fixative was removed, the plates were dried by floating in water bath at 37°C for 20 min. Then 50 μl of formamide was added to each well to denature DNA, and the plates were incubated in an oven at 75°C for 20 min. Afterwards plates were cooled down in refrigerator at 4°C for 5 min. Subsequently, formamide was replaced with 200 μl of 3% non-fat dried milk in each well, and plates were incubated at 37°C for 1 h to block non-specific binding sties. Then each well was replaced with 100 μl of a mixture of primary antibody against single-strand DNA (MAb F7-26) and secondary antibody with peroxidase-conjugated antimouse IgM for 30 min; washed three times with PBS; incubated with peroxidase substrate before detection of absorbance in an ELISA plate reader at 405 nm.

### Statistical analysis

Statistical analyses were performed by the Bonferroni *t*-test method after analysis of variance. A *p* value less than 0.05 was considered significant for all tests. Regression analysis was used to calculate half maximal inhibitory concentration (IC_50_) and 10% inhibitory concentration (IC_10_) values. Data were reported as a mean of 3 experiments with ± standard deviation.

## Results

### Cytotoxic effect of CHM on MKN-74 cell

At first, we studied the cytotoxic effect of 3 commonly used CHM formulas for cancer patients on MKN-74 cells. Cells were treated with CHM for 72 h, and the cell viability was measured by MTT assay. As shown in Table 1, the IC_50_ for BZYQT, BYT and JYJ was 2.4 ± 0.6 mg/ml, 3.9 ± 0.8 mg/ml, and 1.8 ± 0.4 mg/ml, respectively. The IC_10_ for BZYQT, BYT and JYJ was 0.8 ± 0.1 mg/ml, 0.43 ± 0.1 mg/ml, and 0.68 ± 0.1 mg/ml, respectively.

**Table 1:** **Cytotoxicity of chinese herbal medicine on MKN-74 cells**

	**IC**_**10**_ **(mg/ml)**	**IC**_**50**_ **(mg/ml)**
BZYQT	0.8 ± 0.1	2.4 ± 0.6
BYT	0.43 ± 0.0	3.9 ± 0.8
JYJ	0.68 ± 0.1	1.8 ± 0.4

### Effect of CHM on MMC-induced cytotoxicity

**Figure 1:**
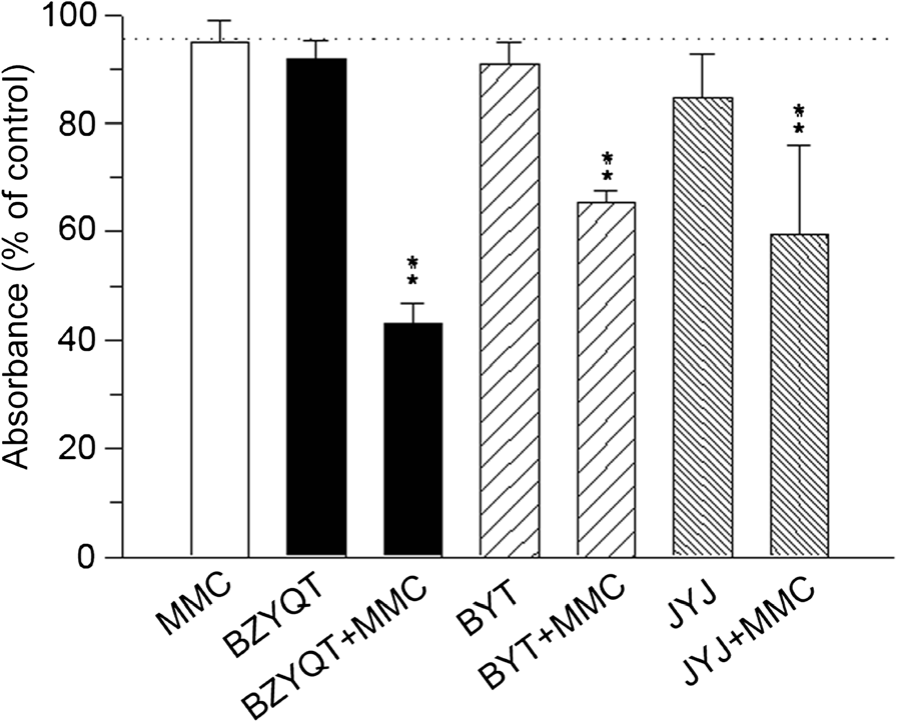
**Effect of chinese herbal medicine formulas on the cytotoxicity induced by MMC.** MKN-74 cells were treated with IC_10_ concentration of 3 Chinese herbal medicine formulas (BZYQT, BYT and JYJ) for 48 h. Then 0.25 μg/ml MMC was added for another 24 h. Cell survival was quantified by MTT assay. Each value was determinate as the percentage of untreated control group. Values were expressed as mean ± SD from three separate experiments. **P <0.01 compared with untreated control group.

MKN-74 cells were treated with PBS as control or various CHM at IC_10_ concentration for 48 h first. Then MMC (0.25 μg/ml) was added to CHM for another 24 h of incubation before assaying for cell viability. This sequence of CHM for 48 h followed by combination of CHM and MMC for 24 h demonstrated significant cytotoxicity on MKN-74 cells. Treatment of BZYQT, BYT or JYJ with MMC resulted in cell viability of 43.1 ± 3.7%, 65.4 ± 2.3%, 59.4 ± 16.5%, respectively; treatment of PBS with MMC showed cell viability of 94.9 ± 3.9% (Figure 1). Among all the CHM tested, BZYQT exhibited the most pronounced effect on the enhancement of MMC-induced cytotoxicity.

### Combination of BZYQT and MMC on the induction of apoptosis

**Figure 2:**
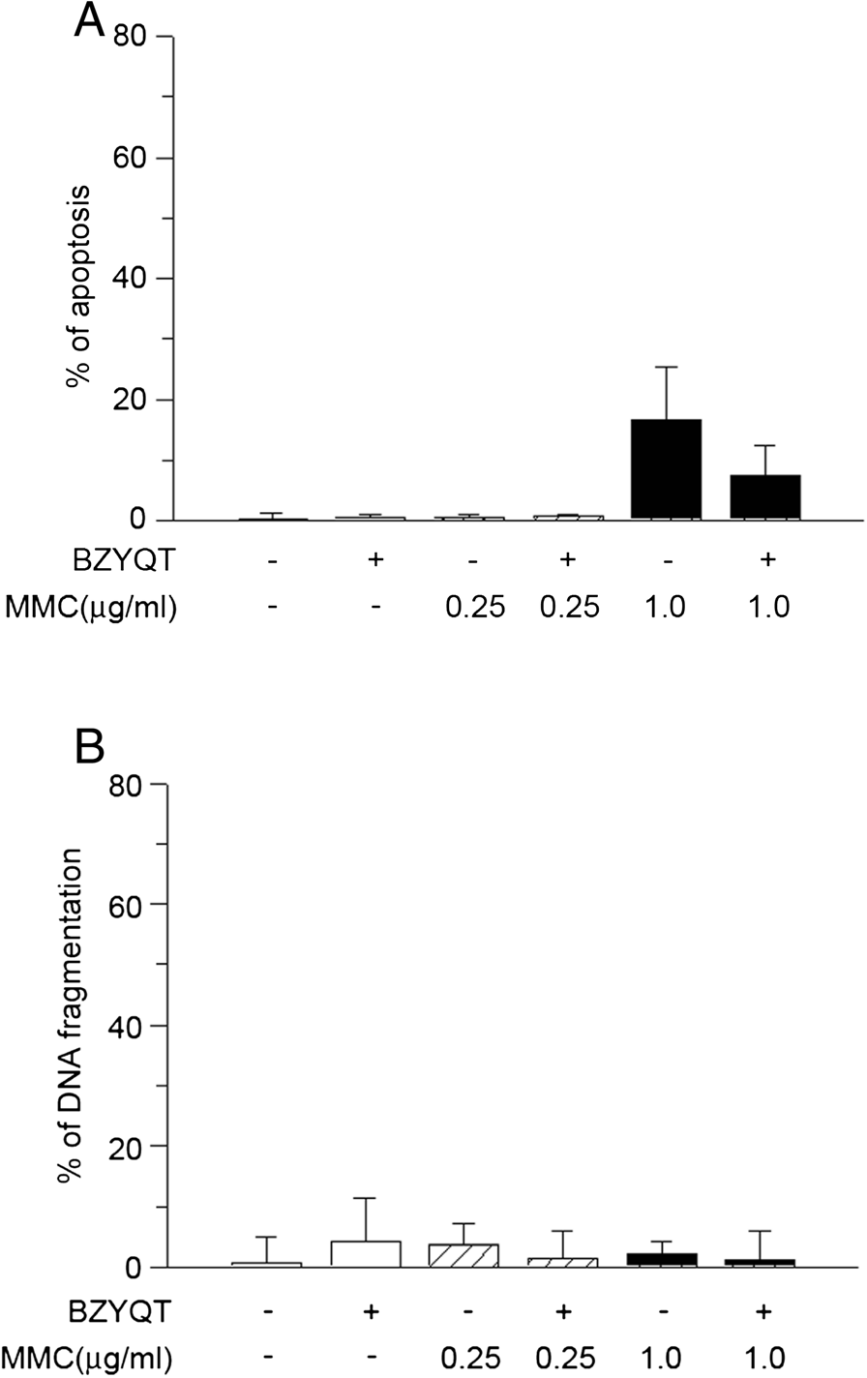
**Effect of combination BZYQT with MMC on induction of apoptosis.** MKN-74 cells were treated with 0.82 mg/ml of BZYQT for 48 h. Then 0.25 or 1.0 μg/ml MMC was added for another 24 h. **(A)** Cells were counted and scored for apoptoic chromatin condensation by the quantitative fluorescence microscopy. Bars represent mean ± SD of cells with apoptosis counted as a percentage of 400 total cells randomly counted in duplicate samples. **(B)** Apoptotic cell death was evaluated by quantitative by DNA fragmentation by using single-strand DNA ELISA kit. Values are expressed as mean ± SD from three separate experiments.

We next assessed whether the enhanced cytotoxicity to MMC by BZYQT was due to apoptosis induction. Apoptosis was evaluated by quantitative fluorescent microscopy of the nuclear changes with bisbenzimide trihydrochloride (Hoechst-33258) staining of condensed nuclear chromatin. MKN-74 cells were treated with PBS or BZYQT for 48 h. Subsequently MMC (either 0.25 or 1.0 μg/ml) was added to the media for another 24 h of incubation. Cells exposed to BZYQT and MMC did not show significant increase in apoptosis induction when compared to PBS and MMC (Figure 2A). Using ELISA for DNA fragmentation as another independent assay for apoptosis measurement, we confirmed the findings from quantitative fluorescent microscopy (Figure 2B). Thus the enhanced cytotoxicity from BZYQT and MMC was not due to induction of apoptoic cell death.

**Figure 3:**
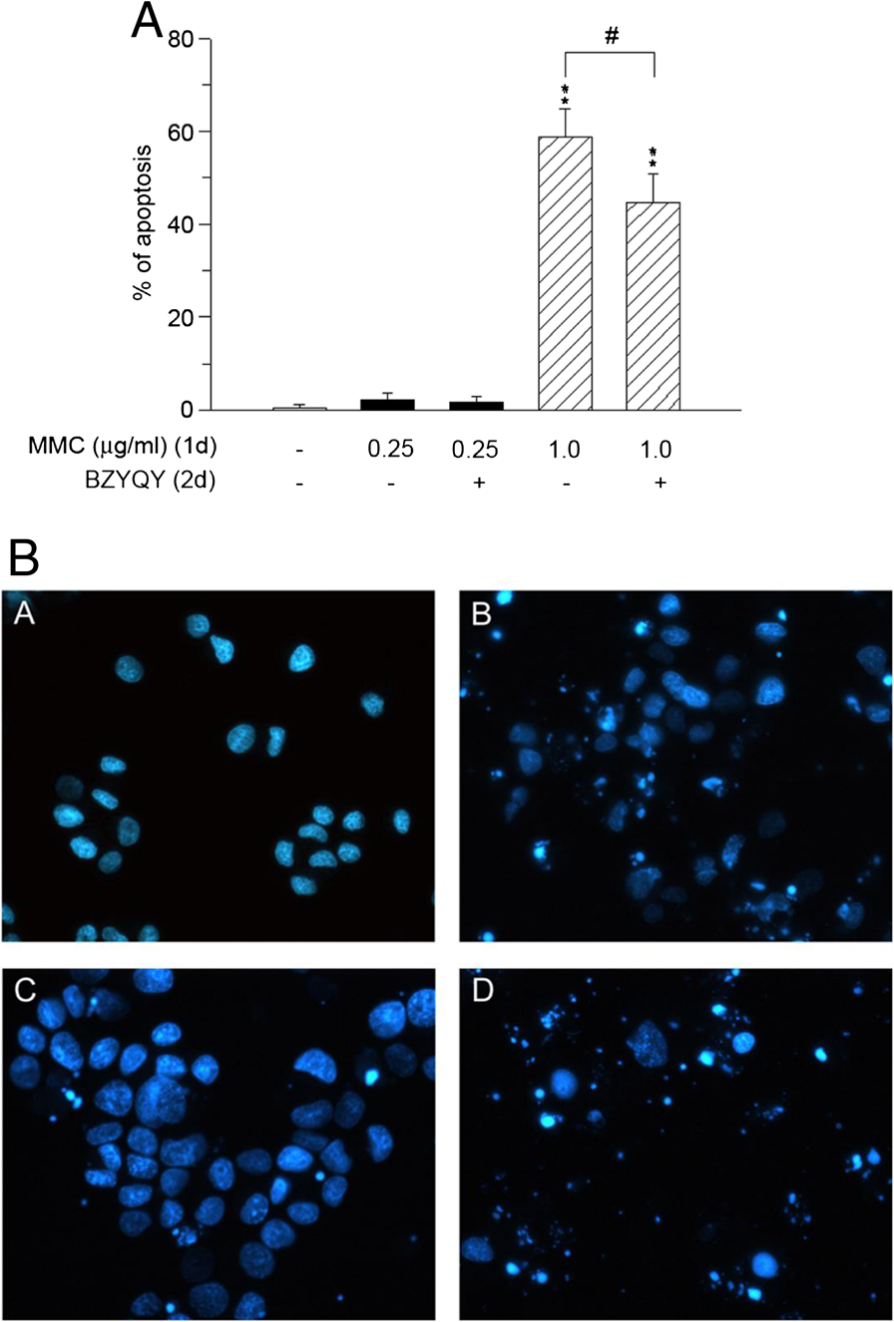
**Effect of pre-treated with MMC followed by exposure to BZYQT on induction of apoptosis. (A)** MKN-74 cells were treated with 0.25 or 1.0 μg/ml MMC for 24 h. Subsequently, cells were washed and treated with or without 0.82 mg/ml of BZYQT for 48 h. Cells were counted and scored for apoptoic chromatin condensation by the quantitative fluorescence microscopy. Bars represent mean ± SD of cells with apoptosis counted as a percentage of 400 total cells randomly counted in duplicate samples. **P <0.01 compared with untreated control group. #*P* < 0.01 compared each other. **(B)** Photomicrographs of representative fields of MKN-74 cells stained with bisbenzimide trihydrochloride (Hoescht-33258) to evaluate nuclear chromatin condensation after treatment with no drug **(panel A),** 1.0 μg/ml MMC for 24 h then cells were washed followed by incubation with no drug for 48 h **(panel B)**, 1.0 μg/ml MMC for 24 h then cells were washed followed by exposure to 0.82 mg/ml of BZYQT for 48 h **(panel C)**, no drug for 48 h prior to 50 μM safingol combined with 1.0 μg/ml MMC for 24 h **(panel D)**.

Moreover, we explored whether the BZYQT could increase MMC-induced apoptosis if cells were treated with MMC for 24 h then washed out with subsequent exposure to BZYQT for another 48 h. As shown in Figure 3A, if cells were exposed to 0.25 μg/ml of MMC for 24 h followed by exposure to BZYQT for another 48 h, there was no significant change in MMC-induced apoptosis. However, in cells treated with 1 μg/ml of MMC followed by exposure to BZYQT for another 48 h, there was a significant decrease in the MMC-induced apoptosis (58.9 ± 5.9% with MMC alone vs. 44.7 ± 6.1% with sequential MMC and BZYQT; *P* < 0.01). The representative photomicrographs are shown in Figure 3B. Safingol, an inhibitor of protein kinase C, which has been shown to enhance MMC-induced apoptosis in human gastric cancer cells, was used as a positive control [15].

## Discussion

In human gastric adenocarcinoma cell, MMC has been shown to induce apoptosis by a caspase-dependent mechanism [16]. Adding PKC inhibitor to MMC further enhances MMC-induced apoptosis [15]. In this study, we explored the effects of commonly used CHM formulas in cancer patients with MMC in human gastric cancer cell line, MKN-74. Among these CHM tested: BZYQT, BYT and JYJ, BZYQT significantly enhanced MMC induced cytotoxicity against MKN-74 cells. Upon further investigation, we confirmed that the enhanced cytotoxicity by BZYQT and MMC was not due to apoptosis induction. Studies done on other CHM formulas such as BYT and JYJ also demonstrated similar results (data not shown). Thus the enhanced cytotoxicity in MKN-74 cells with the combination of CHM and MMC is most likely mediated by necrosis-like cell death.

Necrosis is one type of cell death, which is distinct from apoptosis [17]. During necrosis, cells first swell followed by collapse of plasma membranes collapse, subsequently leading to cell lysis. This process can be induced by various stimuli such as infection, trauma, ischemia and toxin. Apoptosis, on the other hand, is a process of programmed cell death, occurring during physiological process or induced by anti-cancer agents. During apoptosis, there is extensive blebbing of cell membrane followed by chromatin condensation and chromosomal DNA fragmentation.

It has been reported that intracellular ATP level is a determinant factor to induce necrosis-like cell death [18]. Depletion of ATP results in necrotic cell death, and blocks apoptosis signaling [19]. We speculate that treatment of BZYQT in MKN-74 might have led to depletion of intracellular ATP, and then necrotic cell death induced by MMC. BZYQT has been shown to induce apoptosis in human hepatoma cell lines, and the IC_50_ values for different hepatoma cell lines were ranged from 0.43 to 2.28 mg/ml [12]. In this study, the IC_50_ of BZYQT in MKN-74 gastric cancer cell line was 2.4 ± 0.6 mg/ml, which was in the similar range as in the hepatoma cell lines. However, the enhanced MMC-induced cytotoxicity by BZYQT in MKN-74 was not mediated through apoptoic cell death. Thus, the cell death pathway elicited by BZYQT may be cell line dependent. Our findings warrants further confirmation in other cancer cell lines as well in animal studies.

Some compounds in BZYQT such as astragaloside, ginsenoside, saikosaponin and glycyrrhizin have been confirmed antitumor activity [20-23]. It remains to be determined which compound in BZYQT is the active driver for cytotoxicity in gastric cancer cells. It is essential to identify the interaction of CHM with commonly used anticancer therapeutics including biological agents in gastric cancer and other cancer types. In our study, we have found if cancer cells were exposed to MMC first, followed by BZYQT, there were less MMC-induced apoptosis as compared to initial exposure of BZYQT followed by MMC. Thus the sequence of administration schedule for CHM and chemotherapy may alter cytotoxicity and apoptosis induction, and requires further investigation. The findings from this study provide a rationale for further exploration in this aspect.
